# KaKs_Calculator 3.0: Calculating Selective Pressure on Coding and Non-coding Sequences

**DOI:** 10.1016/j.gpb.2021.12.002

**Published:** 2022-01-03

**Authors:** Zhang Zhang

**Affiliations:** 1 National Genomics Data Center, Beijing Institute of Genomics, Chinese Academy of Sciences, Beijing 100101, China; 2 CAS Key Laboratory of Genome Sciences and Information, Beijing Institute of Genomics, Chinese Academy of Sciences, Beijing 100101, China; 3 China National Center for Bioinformation, Beijing 100101, China; 4 University of Chinese Academy of Sciences, Beijing 100049, China

**Keywords:** KaKs_Calculator, Selective pressure, Substitution, Coding, Non-coding

## Abstract

**KaKs_Calculator** 3.0 is an updated toolkit that is capable of calculating **selective pressure** on both **coding** and **non-coding** sequences. Similar to the nonsynonymous/synonymous **substitution** rate ratio for coding sequences, selection on non-coding sequences can be quantified as the ratio of non-coding nucleotide substitution rate to synonymous substitution rate of adjacent coding sequences. As testified on empirical data, KaKs_Calculator 3.0 shows effectiveness to detect the strength and mode of selection operated on molecular sequences, accordingly demonstrating its great potential to achieve genome-wide scan of natural selection on diverse sequences and identification of potentially functional elements at a whole-genome scale. The package of KaKs_Calculator 3.0 is freely available for academic use only at https://ngdc.cncb.ac.cn/biocode/tools/BT000001.

## Introduction

Detecting natural selection on molecular sequences is of fundamental significance in molecular evolution, comparative genomics, and phylogenetic reconstruction, which can provide profound insights for revealing evolutionary processes of molecular sequences and unveiling complex molecular mechanisms of genome evolution [1]. In principle, estimating selection on DNA sequences requires a reference set of substitutions that is free from selection. As synonymous substitutions do not provoke amino acid changes due to the degeneracy of the genetic code, they are expected to be invisible to selection and thus widely used as a reference that reflects the neutral rate of evolution [2]. Consequently, the ratio of nonsynonymous substitution rate (*Ka* or *d_N_*) to synonymous substitution rate (*Ks* or *d_S_*), namely, ω = *Ka*/*Ks* (or *d_N_*/*d_S_*), is widely adopted to differentiate neutral mutation (ω ≈ 1) from negative (purifying) selection (ω < 1) and positive (adaptive) selection (ω > 1), accordingly providing a powerful tool for illuminating molecular evolution of coding sequences (see a popular package in [3]).

Nowadays, a growing body of evidence has shown that non-coding sequences, historically thought as “junk” due to few knowledge on their function relative to coding sequences, are recognized as functional elements to play important regulation roles in multiple biological processes [4] and associate closely with various human diseases [5], [6], [7]. Albeit less conserved by comparison with coding sequences, a larger number of non-coding sequences have been identified highly conserved across mammalian genomes [8], [9], [10]. Importantly, more non-coding sequences are subject to positive selection and negative selection than previously believed, and particularly, long non-coding RNA (lncRNA) sequences do experience natural selection [11]. As a result, several computational methods have been proposed for the detection of selection acting on non-coding sequences [12], which primarily differ in how to choose a reference of unconstrained evolution, such as, synonymous substitutions of neighboring coding gene [13], intron sequences [14], [15], and ancestral repeats [16]. However, there lacks of an implemented algorithm to detect the strength and mode of selective pressure on non-coding sequences, particularly considering an increasing number of non-coding studies conducted worldwide. More importantly, an integrated toolkit that is capable of detecting selection on both coding and non-coding sequences is highly desirable, which would help users achieve genome-wide scan of natural selection on diverse sequences.

Toward this end, here we present KaKs_Calculator 3.0, an updated toolkit for calculating selective pressure on both coding and non-coding sequences. Compared with previous versions [17], [18] that focus solely on coding sequences, we implement an algorithm in KaKs_Calculator 3.0 that employs synonymous sites of adjacent coding sequences as a reference to estimate selective pressure acting on non-coding sequences. We test it on empirical data and demonstrate its utility in diagnosing the strength and form of molecular evolution.

## Algorithm

**Figure 1:**
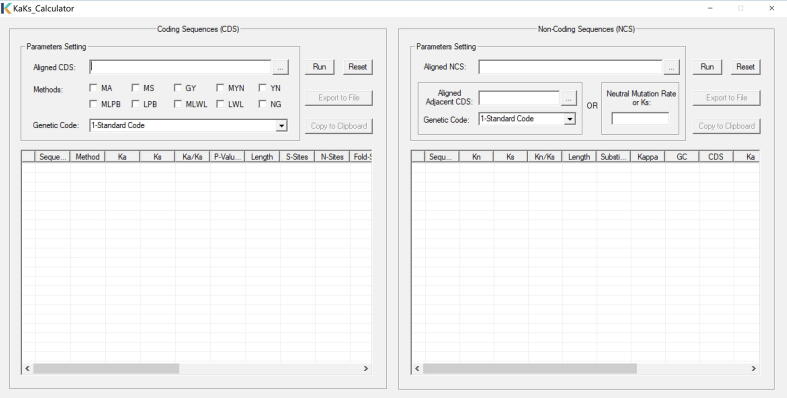
**Graphical user interface of KaKs_Calculator 3.0** It contains two panels that are devised for CDS and NCS, respectively. Methods for detecting selection on CDS are classified as: 1) approximate methods: NG by Nei et al. [23], LWL by Li et al. [22], LPB by Li [24] and Pamilo et al. [29], MLWL and MLPB by Tzeng et al. [28], YN by Yang et al. [26], MYN by Zhang et al. [27]; 2) maximum-likelihood methods: GY by Goldman et al. [25], and MS and MA by Zhang et al. [17]. *Ka*, nonsynonymous substitution rate; *Ks*, synonymous substitution rate; *Kn*, non-coding nucleotide substitution rate; *Ka/Ks*, selective pressure on CDS; *Kn/Ks*, selective pressure on NCS; CDS, coding sequence; NCS, non-coding sequence; MLWL, Modified LWL; MLPB, Modified LPB; MYN, Modified YN; MS, Model Selection; MA, Model Averaging.

The major update of KaKs_Calculator 3.0 is to incorporate an algorithm that is capable of estimating selective pressure on non-coding sequences. Specifically, it uses synonymous substitutions as a reference baseline (similar to [13]), which, albeit thought to be under weak selection [19], [20], [21], has been widely adopted for determining the strength and type of selection operated on coding sequences [22], [23], [24], [25], [26], [27], [28], [29]. Similar to the *Ka*/*Ks* ratio for coding sequences, selective pressure on non-coding sequences (ξ) can be quantified as the ratio of non-coding nucleotide substitution rate (*Kn*) to neutral substitution rate (assumed as *Ks*), *viz.* ξ = *Kn*/*Ks*, where *Ks* is inferred from adjacent coding sequences. As the number of observed substitutions is less than the number of real substitutions, we adopt a nucleotide substitution model (*e.g.*, JC/K2P/HKY) to correct multiple substitutions of non-coding sequences. Taking the HKY model [30] as an example, therefore, *Kn* can be deduced from the observed transitional and transversional substitutions (*S* and *V*, respectively) as well as four nucleotide frequencies (π_A_, π_T_, π_G_, and π_C_) , according to Equation (1) (see Equations 1.27 and 1.28 in [31]).

(1) $$\mathrm{Kn}=2\left(\frac{\pi_{\text{T}}\pi_{\text{C}}}{\pi_{\text{Y}}}+\frac{\pi_{\text{A}}\pi_{\text{G}}}{\pi_{\text{R}}}\right)a-2\left(\frac{\pi_{\text{T}}\pi_{\text{C}}\pi_{\text{R}}}{\pi_{\text{Y}}}+\frac{\pi_{\text{A}}\pi_{\text{G}}\pi_{\text{Y}}}{\pi_{\text{R}}}-\pi_{\text{Y}}\pi_{\text{R}}\right)b$$

where $a=-log[1-\frac{S}{2\left(\pi_{\text{T}}\pi_{\text{C}}/\pi_{\text{Y}}+\pi_{\text{A}}\pi_{\text{G}}/\pi_{\text{R}}\right)}-\frac{\left(\pi_{\text{T}}\pi_{\text{C}}\pi_{\text{R}}/\pi_{\text{Y}}+\pi_{\text{A}}\pi_{\text{G}}\pi_{\text{Y}}/\pi_{\text{R}}\right)V}{2\left(\pi_{\text{T}}\pi_{\text{C}}\pi_{\text{R}}+\pi_{\text{A}}\pi_{\text{G}}\pi_{\text{Y}}\right)}]$, $b=-log(1-\frac{V}{2\pi_{\text{Y}}\pi_{\text{R}}})$, π_R_ = π_A_ + π_G_, and π_Y_ = π_T_ + π_C_. To detect and quantify selection on non-coding sequences, KaKs_Calculator 3.0 provides users with two ways to obtain the value of neutral mutation rate or *Ks*, which is either calculated from adjacent coding sequences uploaded by users or just specified in a straightforward manner by users (Figure 1). As a consequence, KaKs_Calculator 3.0 is capable of detecting selection on both coding and non-coding sequences.

KaKs_Calculator 3.0 is implemented in standard C++ language, enabling higher efficiency and easy compilation on different operation systems (Linux/Windows/Mac). In addition to the new functionality for estimating selection on non-coding sequences as mentioned above, it is also updated by fixing bugs and errors. The package of KaKs_Calculator 3.0, including compiled executables, a Windows application with graphical user interface (GUI), source codes, and example data, accompanying with detailed instructions and documentation, is freely available for academic use only at BioCode (https://ngdc.cncb.ac.cn/biocode/tools/BT000001), an open-source platform for archiving bioinformatics tools in the National Genomics Data Center (NGDC) [32], China National Center for Bioinformation.

## Application on empirical data

To test KaKs_Calculator 3.0, we choose three empirical lncRNA genes that are extensively studied according to LncRNAWiki [7] and collect their human–mouse orthologs as well as their adjacent coding orthologs from NGDC LncBook [33] and National Center of Biotechnology Information (NCBI) RefSeq [34]. Specifically, these non-coding and coding gene symbols with accession numbers are: 1) *H19* (NR_002196.2 *vs*. NR_130973.1) and *MRPL23* (NM_021134.4 *vs*. NM_011288.2); 2) Metastasis-associated lung adenocarcinoma transcript 1 (*MALAT1*; NR_002819.4 *vs*. NR_002847.3) and *SCYL1* (NM_020680.4 *vs*. NM_001361921.1); and 3) *Hox* transcript antisense intergenic RNA (*HOTAIR*; NR_003716.3 *vs*. NR_047528.1) and *HOXC12* (NM_173860.3 *vs*. NM_010463.2). Based on these orthologous genes, we obtain their corresponding aligned sequences by MAFFT [35] (using parameters: --maxiterate 1000 --localpair).

According to the ratio (ξ) of non-coding nucleotide substitution rate to adjacent synonymous substitution rate, we reveal that, although the coding genes undergo strong purifying selection (ω < 1), these three non-coding genes present diverse selective pressure (Table 1). Strikingly, *HOTAIR* exhibits positive selection (ξ > 1), whereas the rest two genes experience negative selection (ξ < 1). *HOTAIR* is a ∼ 2.3-kb intergenic RNA transcribed from the antisense strand of the *HOXC* gene cluster [36]. The result of positive selection detected on *HOTAIR* relative to *HOXC12* is consistent well with previous findings that *HOTAIR* evolves faster than the neighboring genes [37]. On the contrary, *MALAT1*, a ∼ 8.7-kb non-coding RNA flanked by the highly conserved kinase-like gene *SCYL1*, is ubiquitously expressed in almost all human tissues, evolutionarily conserved across mammalian species [38], and associated with various cancers [39]. Thus, ξ = 0.464 indicates strong selective constraint on *MALAT1*, in accordance with its physiologic and pathophysiological function [40] and conserved RNA structure [41] as documented by previous studies. Likewise, *H19*, a ∼ 2.3-kb imprinted maternally expressed transcript located near *MRPL23*, is known for close association with Beckwith-Wiedemann Syndrome and also involved in tumorigenesis [42]. Our result shows that *H19* presents stronger selection constraint as indicated by ξ = 0.296, conforming well with its conserved sequence and structure [43]. It is worth noting that one non-coding sequence may have multiple adjacent coding genes, which are specified by users and thus can lead to different estimates of *Ks* and ξ. Taken together, KaKs_Calculator 3.0 is effective in estimating natural selection on non-coding sequences, which has the potential to reveal evolutionarily selective pressures operated on diverse molecular sequences.

**Table 1:** **Estimates of selective pressure as well as substitution rates in human–mouse orthologs**

**Non-coding**			**Coding**			
**Gene symbol**	***Kn***	**ξ** **=** ***Kn*/*Ks***	**Gene symbol**	***Ka***	***Ks***	**ω** **=** ***Ka*/*Ks***
*H19*	0.340	0.296	*MRPL23*	0.088	1.150	0.077
*MALAT1*	0.324	0.464	*SCYL1*	0.040	0.697	0.058
*HOTAIR*	0.544	1.114	*HOXC12*	0.020	0.488	0.041

*Note*: *Ka*, nonsynonymous substitution rate; *Ks*, synonymous substitution rate; *Kn*, non-coding nucleotide substitution rate; ω, selective pressure on coding sequence; ξ, selective pressure on non-coding sequence.

In addition, to test the running performance of KaKs_Calculator, we collect an empirical large dataset that contains 15,424 human–mouse orthologous genes retrieved from RefSeq [34] and obtain their codon-based alignments by ParaAT [44] — a parallel tool for constructing multiple protein-coding DNA alignments. KaKs_Calculator 3.0 includes ten computational methods for detecting selection on coding sequences, which fall into approximate methods and maximum-likelihood methods. We choose three approximate methods, NG [23], YN [26], and MYN [27], and one maximum-likelihood method, GY [25], and test on a 64 bit x86 Intel Core i7 machine containing 4 CPU cores with each 3.40 GHz and running Windows 10. For this large-scale data analysis, we find that NG, YN, and MYN all take ∼ 2 min and GY takes ∼ 11 h, clearly showing that approximate methods are more time-efficient than maximum-likelihood ones. Considering that different users may have different preferences, it should be noted, however, that maximum-likelihood methods are believed to achieve higher accuracy and that different methods adopt different models and strategies and thus can lead to different estimates [45] (see an example in [46] where contradictory findings are produced by different methods).

## Discussion

KaKs_Calculator 3.0 is significantly updated by achieving the detection of natural selection on non-coding sequences as well as coding sequences. As testified on empirical data, it is of great utility in calculating natural selection on molecular sequences, thus identifying potentially functional elements at a genome-wide scale. Future developments include the detection of selective pressure on small peptides (less than 300 nucleotides) that are encoded by small open reading frames within non-coding sequences [47], [48], [49] as well as the implementation of codon-based alignment procedure to help users generate input sequences in an easy-to-use manner.

## Code availability

KaKs_Calculator 3.0 is freely available for academic use only at https://ngdc.cncb.ac.cn/biocode/tools/BT000001.

## CRediT authorstatement

**Zhang Zhang:** Conceptualization, Methodology, Software, Writing - original draft, Writing - review & editing, Funding acquisition, Supervision. The author has read and approved the final manuscript.

## Competing interests

The author has declared no competing interests.
